# Incredibly late thromboses in first generation drug eluting stents: a case series

**DOI:** 10.1186/1757-1626-2-9303

**Published:** 2009-12-10

**Authors:** Zakir Shaik, Khaja S Mohammed, Adnan Siddiqi, Ilyas Mohammed, Timothy A Shapiro, Thomas P Phiambolis

**Affiliations:** 1 Department of Internal Medicine, Main Line Health, Lankenau Hospital, 100 Lancaster Road, Wynnewood, Pennsylvania 19096, USA; 2 Division of Cardiology, Main Line Health, Lankenau Hospital, 100 Lancaster Road, Wynnewood, Pennsylvania 19096, USA

## Abstract

**Background:**

The drug-eluting stents have decreased the incidence of instent restenosis compared to bare metal stents. But, the incidence of late and very late stent thrombosis has increased with the drug-eluting stents.

**Case presentation:**

We are here, reporting three cases of incredibly late instent thrombosis, each one occurring after more than 50 months of drug-eluting stent placement.

**Conclusion:**

The occurrence of stent thrombosis as late as 5 years has been reported in literature. This highlights the importance that there may be no limit to the time duration to the occurrence of very late stent thrombosis and dual antiplatelet therapy with aspirin and clopidogrel may have to be continued indefinitely in patients with drug-eluting stents.

## Background

Though drug-eluting stents (DES) when compared with bare metal stents (BMS) have decreased the incidence of instent restenosis and subsequent target vessel revascularization, studies have highlighted the increased incidence of late and very late stent thromboses (VLST) with DES and their potential adverse clinical outcomes.

As per the standard definition from the Academic Research Consortium (ARC) stent thrombosis (ST) can be called acute, subacute, late and very late based on the timing [1]. An acute ST is defined as ST during percutaneous coronary intervention (PCI) or within the following 24 hours, subacute ST as between 1 and 30 days following PCI, late ST as between 1 month and 1 year following the PCI, and very late ST as more than one year following the PCI. The ARC has also standardized the definitions of a definite ST, possible ST and probable ST based on different criteria.

The longest reported interval between the implantation and thrombosis of a DES is about 67 months [2]. Here we report three cases of incredibly late ST occurring after 51 months (case 1), 58 months (case 2) and more than 50 months (case 3) from implantation, respectively. To our knowledge, the duration of 58 months in one of our cases represents one of the longest reported latencies from the placement to the occurrence of thrombosis described in the literature for the DES.

## Case presentation

### Case report 1

A 60 year old Caucasian male presented with an acute coronary syndrome in October 2003. He had a history of hypertension, tobacco use, dyslipidemia and a family history of coronary artery disease. The coronary angiogram (CAG) showed 80% focal stenosis in the distal portion of the right coronary artery (RCA). The lesion was treated with a 2.5 × 18 mm sirolimus eluting coronary stent (Cypher stent, Johnson & Johnson, New Brunswick, New Jersey, USA) deployed at 12 atmospheres and post dilated at 14 atmospheres with a 2.75 mm non-compliant balloon. There was TIMI (Thrombolysis in myocardial infarction) grade 3 flow with no complications. He was discharged on aspirin and clopidogrel along with other standard medical regimen. While continuing to take the dual anti-platelet therapy he was readmitted on November 2004 with unstable angina. The CAG at this time showed a patent distal RCA stent but the posterior descending artery (PDA) was critically narrowed. The PDA lesion was treated with a 2.5 × 13 mm sirolimus eluting stent (Cypher stent, Johnson & Johnson, New Brunswick, New Jersey, USA) deployed at 11 atmospheres with satisfactory results and no complications. He was once again discharged on dual anti-platelet therapy.

**Figure 1:**
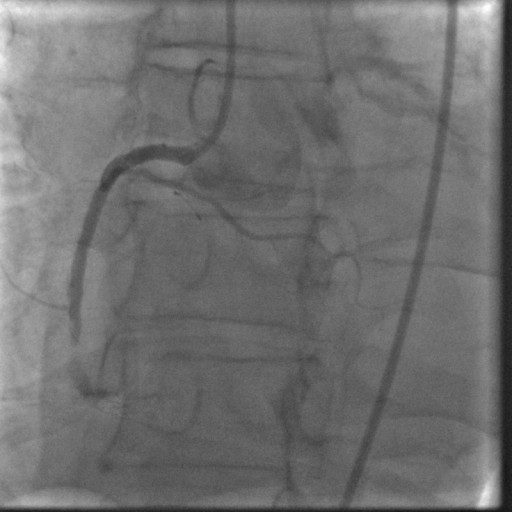
**Angiogram showing Thrombotic occlusion of DES in distal Right Coronary Artery**.

**Figure 2:**
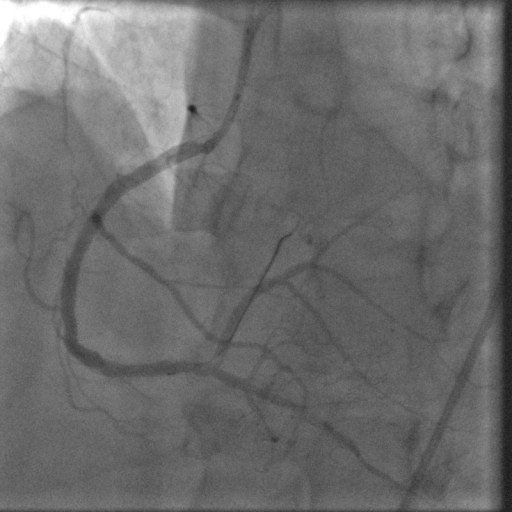
**Angiogram of the distal Right Coronary Artery lesion after standard PCI**.

In January 2008, the patient was readmitted with an acute inferior wall ST-elevation myocardial infarction (STEMI). He had continued on aspirin and clopidogrel since his second stent was placed. His aspirin and clopidogrel were held 10 days prior to this episode for a dental procedure. On admission an emergent cardiac catheterization was performed which showed a thrombotic total occlusion of the previously placed DES in the distal RCA (Figure 1). The lesion was successfully dealt with using standard percutaneous intervention procedures (Figure 2). The post-interventional course was uneventful and the patient was discharged on dual anti-platelet therapy (DAT) WITH aspirin 325 mg and clopidogrel 75 mg, to be continued indefinitely.

### Case report 2

A 66 year old Caucasian male with the past history of hypertension and dyslipidemia underwent a cardiac catheterization in December 2003 because of exertional angina and a positive stress test. It showed critical disease with 90% stenoses of both the mid left anterior descending artery (LAD) and the mid RCA. The sirolimus eluting coronary stents (Cypher stent, Johnson & Johnson, New Brunswick, New Jersey, USA) 2.75 × 18 mm and 2.5 × 18 mm were deployed at 17 atmospheres in both the mid LAD and RCA lesions, respectively. Using a Quantum Maverick 3.0 × 12 mm balloon, both were post-dilated at 18 atmospheres with a 0% residual stenosis, TIMI grade 3 flow and no peri-procedural complications. He was subsequently discharged on aspirin, clopidogrel and other standard medical regimen. The clopidogrel was stopped after the recommended treatment period of 3 months and after which he was continued on aspirin alone.

**Figure 3:**
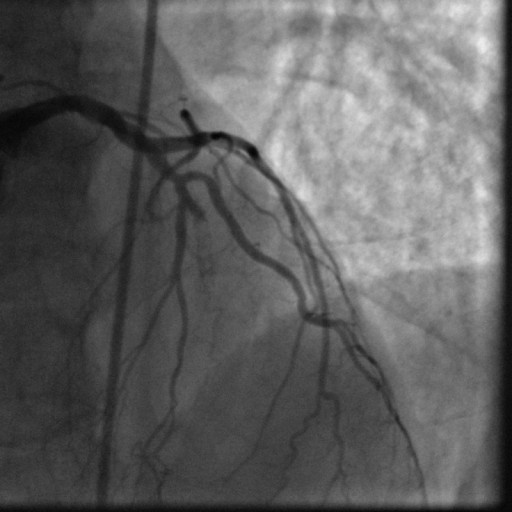
**Angiogram showing complete thrombosis of DES in mid Left Anterior Descending artery**.

**Figure 4:**
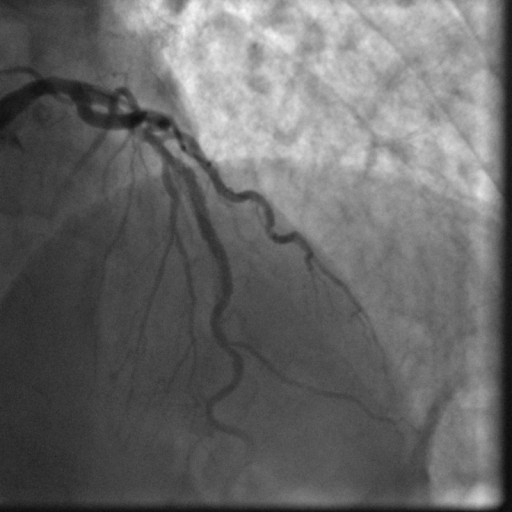
**Angiogram of mid Left Anterior Descending Artery lesion after Standard PCI**.

In October 2008, nearly 5 years post-stent placement, the patient was readmitted with an acute antero-septal ST-elevation MI. His aspirin was held about a week prior to this episode for a colonoscopy. An emergent left heart catheterization was performed which revealed a 100% occlusion of the mid LAD stent secondary to thrombosis and a 70% to 80% stenosis of the RCA within the stent secondary to likely thrombus (Figure 3). The patient was loaded with 600 mg clopidogrel and abciximab infusion was started prior to the procedure. Both the lesions were dealt with successfully using standard procedural techniques with 0% residual stenosis and TIMI III flow (Figure 4). The 2D echocardiogram showed an akinetic left ventricular antero-apical region consistent with a LAD territory infarct and an ejection fraction of 35% to 40%. The post-interventional course was uneventful and the patient was discharged on aspirin 325 mg and clopidogrel 75 mg, to be taken indefinitely.

### Case report 3

A 69 year old Caucasian male with a past history of hypertension, type 2 diabetes, dyslipidemia, coronary artery disease, history of coronary artery bypass graft to LAD, RCA and diagonal arteries in 1998, underwent an elective PCI with a paclitaxel-eluting coronary stent (Taxus Stent, Boston Scientific, Massachusetts, USA) to the proximal left circumflex artery in the early 2004 at another hospital. Our access to those records was limited. The patient was continued on DAT with aspirin and clopidogrel for nearly 6 months post-stent implantation following which clopidogrel was discontinued on account of extensive bruising.

**Figure 5:**
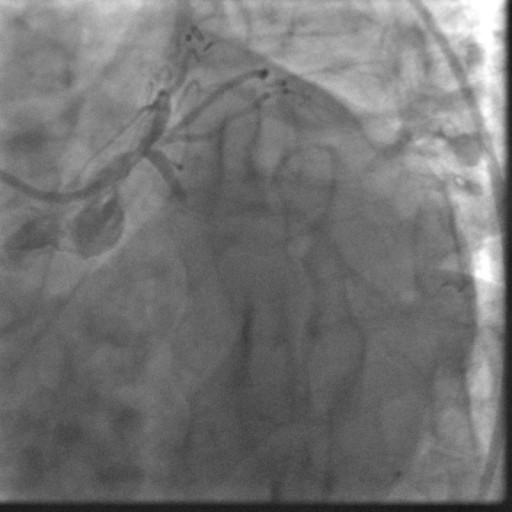
**Angiogram showing thrombosis of DES in Left Circumflex artery**.

**Figure 6:**
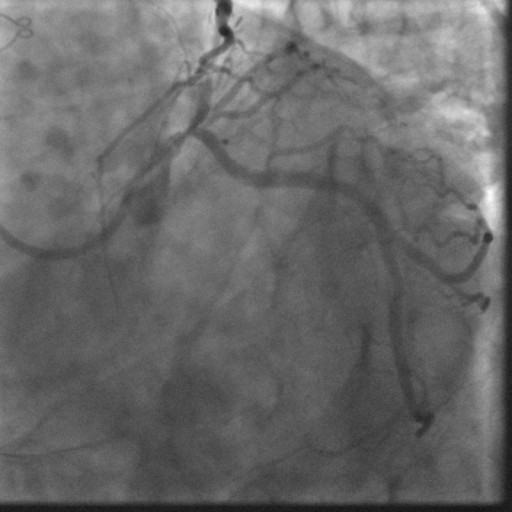
**Angiogram of Left Circumflex after standard PCI**.

More than 4 years later, in December 2008, the patient was admitted to our hospital with acute retrosternal chest pain associated with nausea and vomiting. The EKG showed sinus rhythm with an old left bundle branch block. The biomarkers were positive with a troponins I of 39.9 and a creatinine phosphokinase (CPK) of 1259. He underwent an emergent cardiac catheterization that revealed patent bypass grafts but also showed a thrombus in the previously placed stent in the proximal left circumflex with a 99% angiographic stenosis and TIMI 1 to 2 flow (Figure 5). The lesion was successfully treated with a percutaneous transluminal coronary angioplasty including mechanical thrombectomy using an X-Port catheter and using a Quantum Maverick 3.5 × 18 mm balloon with restoration of TIMI 3 flow and no significant residual stenosis or evidence of distal embolization (Figure 6). The post-interventional course was uneventful and the patient was discharged on DAT.

## Conclusion

The very late stent thrombosis (VLST) which occurs more than one year after stent implantation is a serious complication of first generation DES. The VLST has been reported to occur at rates varying between 0.2 to 0.7% of the DES-treated patients in various studies [3-7]. However, the incidence of VLST may be higher in real life than in clinical trials because the real-life patients are potentially sicker, their lesions may be more complex, and follow up is longer.

The etiology of ST with DES is not yet completely elucidated. The discontinuation of thienopyridine therapy was shown to be a major determinant of ST within the first 6 months but discontinuation after 6 months did not predict the occurrence of ST in one study (hazard ratio 0.94) [8]. Several other determinants including renal failure, diabetes, low ejection fraction, in-stent restenosis, bifurcation lesions, type C lesions, and a trend for smaller-diameter stents have also been suggested to be the significant correlates of late stent thrombosis [6,9]. Besides these risk factors, delayed arterial healing post-stent implantation in combination with other risk factors like local hypersensitivity reaction, ostial location, mal-apposition or incomplete apposition of the stent and strut penetration into a necrotic lipid core have all been suggested to play a role in late stent thrombosis [10]. The cases of single DES thrombosis in patients with multiple intracoronary DES of the same duration suggest that the factors like incomplete stent apposition and/or, under-expansion and/or, significant residual reference-segment disease play a considerable role in the pathogenesis.

Pathologically, incomplete neointimal coverage leading to a delayed arterial healing in DES is probably an important explanation for the increased risk of thrombosis (especially late or very late ST) in DES compared to BMS. A serial angioscopic follow up study comparing BMS and DES group of patients demonstrated that neointimal coverage was complete in all patients with BMS at ten months while the majority of patients with DES (sirolimus stents) had incomplete coverage at 21 months (P < 0.05) [11]. It has also shown that the mural red thrombi were seen only in patients with DES and in those with incomplete neointimal coverage. From a registry study of 40 autopsies of DES (68 stents) by Joner et al, 23 DES cases of more than 30 days duration were compared with 25 matched autopsies of BMS implantation [10]. Out of those 23 patients with DES more than 30 days old, 14 had evidence of late stent thromboses and the delayed arterial healing was more pronounced in DES patients with late stent thrombosis than in those without stent thrombosis. In the same study, DES also showed poorer endothelialization (55.8 +/- 26.5%) compared with BMS (89.8 +/- 20.9, p = 0.0001).

Ever since the approval of DES in 2003, cases of very late ST with increasing time interval between the implantation of DES and the thrombotic events are being reported. To our knowledge, the duration of 58 months in one of our cases represents one of the longest latency from implantation to thrombosis described in the literature for the DES. More and more reporting of such cases is extremely important to unravel the extent of the risk. These cases highlight the fact that there may be no limit to the time period for the definition of VLST, especially upon cessation of antiplatelet therapy. The optimal duration of antiplatelet therapy (whether single or dual) remains questionable although the 2007 ACC/AHA/SCAI focused update of the 2005 PCI guidelines recommended clopidogrel 75 mg daily for at least 12 months in patients who receive a DES [12].

The guidelines might have to be revised recommending continuation of antiplatelet therapy for an unlimited period of time (even lifelong) for patients with a DES. The newer antiplatelet therapy like prasugrel (TRITON TIMI 38) also may affect treatment algorithm [13]. More importantly, before implanting a DES in any patient (either on-label or off-label use), strong considerations should be given to the degree of risk for restenosis and the patient's ability to comply with long-term dual antiplatelet therapy. DES implantation should be avoided if there is any doubt that the patient can comply with prolonged dual antiplatelet therapy and if the likelihood of future bleeding risk or necessity for surgical/invasive procedures is high.

Even if recommended to continue the antiplatelet therapy indefinitely in DES patients, the issue of incomplete neointimal coverage after a long term period with DES remains unresolved and little is known whether continuing the antiplatelet therapy itself will counterbalance the risks of having exposed stent struts, as cases of very late ST have also occurred while the DES patients were taking uninterrupted dual antiplatelet therapy [14]. However, 34 out of 36 cases of late ST in DES in one analysis were without clopidogrel at the time of the event [15]. It makes a stronger argument for continued long term DAT in such patients. Larger randomized studies with longer follow-ups are needed to answer these questions with certainty. The new generation DES may behave differently and likely to have an improved safety profile. But, the data is very limited at this time and there is a need for large registries, clinical trials and head to head comparisons in a variety of situations before we conclusively prove they have greater efficacy and improved safety.

## Consent

Written informed consent was obtained from each patient for publication of this case series and accompanying images. A copy of the written consent is available for review by the Editor-in-Chief of this journal.

## Competing interests

The authors declare that they have no competing interests.

## Authors' contributions

ZS, KM, and IM drafted and wrote the manuscript. AS, TS, and TP participated in the care of the patients. All authors read and approved the final manuscript.

## References

[B1] Cutlip DE Windecker S Mehran R Boam A Cohen DJ van Es GA Steg PG Morel MA Mauri L Vranckx P McFadden E Lansky A Hamon M Krucoff MW Serruys PW Clinical end points in coronary stent trials: a case for standardized definitions Circulation 2007 115 2344 2351 10.1161/CIRCULATIONAHA.106.685313 17470709

[B2] Layland J Jellis C Whitbourn R Extremely late drug-eluting stent thrombosis: 2037 days after deployment Cardiovasc Revasc Med 2009 10 1 55 57 10.1016/j.carrev.2008.05.001 19159856

[B3] Ong AT McFadden EP Regar E de Jaegere PP van Domburg RT Serruys PW Late angiographic stent thrombosis (LAST) events with drug-eluting stents J Am Coll Cardiol 2005 45 12 2088 2092 10.1016/j.jacc.2005.02.086 15963413

[B4] Urban P Gershlick AH Guagliumi G Guyon P Lotan C Schofer J Seth A Sousa JE Wijns W Berge C Deme M Stoll HP Safety of coronary sirolimus-eluting stents in daily clinical practice: one-year follow-up of the e-Cypher registry Circulation 2006 113 11 1434 1441 10.1161/CIRCULATIONAHA.104.532242 16534015

[B5] Iakovou I Schmidt T Bonizzoni E Ge L Sangiorgi GM Stankovic G Airoldi F Chieffo A Montorfano M Carlino M Michev I Corvaja N Briguori C Gerckens U Grube E Colombo A Incidence, predictors, and outcome of thrombosis after successful implantation of drug-eluting stents JAMA 2005 293 17 2126 2130 10.1001/jama.293.17.2126 15870416

[B6] Stone GW Moses JW Ellis SG Schofer J Dawkins KD Morice MC Colombo A Schampaert E Grube E Kirtane AJ Cutlip DE Fahy M Pocock SJ Mehran R Leon MB Safety and efficacy of sirolimus- and paclitaxel-eluting coronary stents N Engl J Med 2007 356 998 1008 10.1056/NEJMoa067193 17296824

[B7] Kastrati A Mehilli J Pache J Kaiser C Valgimigli M Kelbaek H Menichelli M Sabate M Suttorp MJ Baumgart D Seyfarth M Pfisterer ME Schomig A Analysis of 14 trials comparing sirolimus-eluting stents with bare-metal stents N Engl J Med 2007 356 1030 1039 10.1056/NEJMoa067484 17296823

[B8] Airoldi F Colombo A Morici N Latib A Cosgrave J Buellesfeld L Bonizzoni E Carlino M Gerckens U Godino C Melzi G Michev I Montorfano M Sangiorgi GM Qasim A Chieffo A Briguori C Grube E Incidence and predictors of drug-eluting stent thrombosis during and after discontinuation of thienopyridine treatment Circulation 2007 116 7 745 54 10.1161/CIRCULATIONAHA.106.686048 17664375

[B9] Kuchulakanti PK Chu WW Torguson R Ohlmann P Rha SW Clavijo LC Kim SW Bui A Gevorkian N Xue Z Smith K Fournadjieva J Suddath WO Satler LF Pichard AD Kent KM Waksman R-Correlates and long-term outcomes of angiographically proven stent thrombosis with sirolimus- and paclitaxel-eluting stents Circulation 2006 113 8 1108 1113 10.1161/CIRCULATIONAHA.105.600155 16490815

[B10] Joner M Finn AV Farb A Mont EK Kolodgie FD Ladich E Kutys R Skorija K Gold HK Virmani R Pathology of drug-eluting stents in humans: delayed healing and late thrombotic risk J Am Coll Cardiol 2006 48 1 193 202 10.1016/j.jacc.2006.03.042 16814667

[B11] Awata M Kotani J Uematsu M Morozumi T Watanabe T Onishi T Iida O Sera F Nanto S Hori M Nagata S Serial angioscopic evidence of incomplete neointimal coverage after sirolimus-eluting stent implantation: comparison with bare-metal stents Circulation 2007 116 8 910 916 10.1161/CIRCULATIONAHA.105.609057 17684153

[B12] King SB Smith SC Jr Hirshfeld JW Jr Jacobs AK Morrison DA Williams DO 2005 WRITING COMMITTEE MEMBERS Feldman TE Kern MJ O'Neill WW Schaff HV Whitlow PL Adams CD Anderson JL Buller CE Creager MA Ettinger SM Halperin JL Hunt SA Krumholz HM Kushner FG Lytle BW Nishimura R Page RL Riegel B Tarkington LG Yancy CW 2007 focused update of the ACC/AHA/SCAI 2005 guideline update for percutaneous coronary intervention: a report of the American College of Cardiology/American Heart Association task force on practice guidelines: 2007 writing group to review new evidence and update the ACC/AHA/SCAI 2005 guideline update for percutaneous coronary intervention, writing onbehalf of the 2005 writing committee Circulation 2008 117 261 10.1161/CIRCULATIONAHA.107.188208 18079354

[B13] Wiviott SD Braunwald E McCabe CH Montalescot G Ruzyllo W Gottlieb S Neumann FJ Ardissino D De Servi S Murphy SA Riesmeyer J Weerakkody G Gibson CM Antman EM Prasugrel versus clopidogrel in patients with acute coronary syndromes N Engl J Med 2007 357 20 2001 2015 10.1056/NEJMoa0706482 17982182

[B14] Lim SY Kim KS Joo SJ Jeong MH Very late stent thrombosis after drug-eluting stent implantation in a patient without aspirin and clopidogrel resistance J Korean Med Sci 2008 23 3 556 559 10.3346/jkms.2008.23.3.556 18583901 PMC2526514

[B15] Artang R Dieter RS Analysis of 36 reported cases of late thrombosis in drug-eluting stents placed in coronary arteries Am J Cardio 2007 99 8 1039 1043 10.1016/j.amjcard.2006.12.025 17437724

